# NEIGHBORHOOD CONTEXT AND QUALITY OF LIFE OF OLDER ADULTS IN EASTERN NEPAL: A CROSS-SECTIONAL STUDY

**DOI:** 10.1093/geroni/igad104.1159

**Published:** 2023-12-21

**Authors:** Krishna Sapkota, Saruna Ghimire, Aman Shrestha

**Affiliations:** Miami University, Oxford, Ohio, Oxford, Ohio, United States; Miami University, Oxford, Ohio, United States; Miami University, Oxford, Ohio, United States

## Abstract

**Introduction:**

Increased life expectancy has resulted in population aging and subsequently entails addressing the needs of older adults. Shifting family structures from multigenerational to nuclear have had a significant effect on the older adults’ livelihood and their ways of interaction with community members. This research aims to comprehend the relationship between neighborhood context and older adults’ quality of life (QoL) in Nepal.

**Methods:**

A total of 847 non-institutionalized older adults (≥60 years) from eastern Nepal were interviewed in 2021. The QoL was measured by13-item version of the Older People’s Quality of Life questionnaire (OPQOL-brief), and each item was measured on a 5-point Likert scale. The OPQoL mean score < 3 was recoded as low, and ≥3 was high QoL. Ethnic diversity, monthly income, connection with family, friends, and neighbors, cultural connection, residential instability, and accessibility were measured to see the effect of neighborhood context. Multivariable logistic regression assessed the relationship between neighborhood context and QoL.

**Results:**

More than 20% of older adults reported low QoL. Participants engaged in occupation [OR=2.63], not consuming tobacco [OR=1.78], living in communities with higher ethnic diversity [OR=1.03], frequently in contact with family [OR=4.77] and neighbor [OR=13.79], and stable residence [OR=5.43] had significant associations with better QoL.

**Conclusion:**

In this study, neighborhood context was a determinant of better QoL in older adults. Therefore, engagement in income generation activities, and strengthening families and neighborhood ties bring good QoL among older adults.

